# Circulation of pantropic canine coronavirus in autochthonous and imported dogs, Italy

**DOI:** 10.1111/tbed.13542

**Published:** 2020-04-05

**Authors:** Flora Alfano, Giovanna Fusco, Viviana Mari, Leonardo Occhiogrosso, Gianluca Miletti, Roberta Brunetti, Giorgio Galiero, Costantina Desario, Margie Cirilli, Nicola Decaro

**Affiliations:** ^1^ Istituto Zooprofilattico Sperimentale del Mezzogiorno Portici (Napoli) Italy; ^2^ Dipartimento di Medicina Veterinaria Università degli Studi di Bari Valenzano (Bari) Italy

**Keywords:** animal importation, dog, pantropic canine coronavirus, viral co‐infections

## Abstract

Canine coronavirus (CCoV) strains with the ability to spread to internal organs, also known as pantropic CCoVs (pCCoVs), have been detected in domestic dogs and wild carnivores. Our study focused on the detection and molecular characterization of pCCoV strains circulating in Italy during the period 2014–2017 in autochthonous dogs, in dogs imported from eastern Europe or illegally imported from an unknown country. Samples from the gut and internal organs of 352 dogs were screened for CCoV; putative pCCoV strains, belonging to subtype CCoV‐IIa, were identified in the internal organs of 35 of the examined dogs. Fifteen pCCoV strains were subjected to sequence and phylogenetic analyses, showing that three strains (98960‐1/2016, 98960‐3/2016, 98960‐4/2016) did not cluster either with Italian or European CCoVs, being more closely related to alphacoronaviruses circulating in Asia with which they displayed a 94%–96% nucleotide identity in partial spike protein gene sequences. The pCCoV‐positive samples were also tested for other canine viruses, showing co‐infections mainly with canine parvovirus.

## 1 INTRODUCTION

Coronaviruses (CoVs) are viruses that infect a variety of mammals, including humans, and birds. Coronaviruses (CoVs, order *Nidovirales*, family *Coronaviridae*) are enveloped, single‐stranded, positive‐sense RNA viruses, commonly associated with mild enteritis and/or respiratory signs (Decaro et al., 2013). The family *Coronaviridae* is organized into two subfamilies, *Orthocoronavirinae* and *Letovirinae*, with the former including four different genera, *Alphacoronavirus*, *Betacoronavirus*, *Gammacoronavirus* and *Deltacoronavirus*. Canine coronavirus (CCoV) belongs to the genus *Alphacoronavirus* and forms a unique species, *Alphacoronavirus‐1*, along with feline coronaviruses (FCoVs), transmissible gastroenteritis virus of swine (TGEV), and its derivative, porcine respiratory coronavirus (PRCoV) (Decaro & Buonavoglia, 2008, 2011).

Canine coronavirus infection is restricted to the enteric tract and causes mild or asymptomatic forms of enteritis. Canine coronavirus includes two genotypes: CCoV‐I and CCoV‐II, which share up to 96% of nucleotide (nt) sequence identity in the viral genome, but are highly divergent in the spike (S) protein gene. CCoV‐II is classified into two subtypes: the classical CCoV‐IIa and the recombinant CCoV‐IIb (Decaro et al., 2009).

A hypervirulent CCoV‐IIa strain, referred to as pantropic CCoV (pCCoV), was isolated in Italy in 2005 (Buonavoglia et al., 2006) and is able to spread to extraintestinal tissues. This variant was associated with a fatal disease of dogs, characterized by leukopenia, gastroenteritis and severe lesions in the major organs (Buonavoglia et al., 2006; Chen et al., 2019; Decaro et al., 2008; Pinto et al., 2014), and subsequent studies have proved its impact on canine immune response (Marinaro et al., 2010).

Currently, there are no diagnostic tools capable to differentiate the pantropic strains from the enteric CCoVs, since they are strictly related at the genetic and antigenic level and no specific marker of pathogenicity has been detected so far (Decaro et al., 2013). Thus, the identification of pCCoV relies on the detection of CCoV‐IIa in extraintestinal tissues. To date, pCCoV infection has been reported mainly in dogs (Chen et al., 2019; Decaro et al., 2012, 2013; Ntafis et al., 2012; Pinto et al., 2014; Zicola et al., 2012), although a hypervirulent CCoV‐IIa strain was also reported in a wolf (Alfano et al., 2019). In most cases pCCoV was found in association with co‐pathogens, including canine parvovirus (CPV) (Alfano et al., 2019; Decaro et al., 2012, 2013; Ntafis et al., 2012; Pinto et al., 2014; Zicola et al., 2012) and canine adenovirus type 1 (CAdV‐1) (Alfano et al., 2019).

Taking into account the scarce information existing about the actual circulation of pCCoV in the dog population, the aims of our study were the following: (a) to conduct an epidemiological survey for this virus in autochthonous and imported dogs in Italy during years 2014–2017; (b) to investigate the genetic relatedness of the detected pCCoV strains to extant coronaviruses; and (c) to evaluate the presence of co‐infections in pCCoV positive and negative samples.

## 2 MATERIALS AND METHODS

### 2.1 Samples collection

During the period 2014–2017, 2,112 necropsy samples collected from different tissues (brain, heart, intestine, liver, spleen, lungs, kidney) of 352 dogs were submitted to molecular analysis to investigate possible viral causes of disease. The sampled animals included 141 client‐owned, 151 stray and 60 imported dogs. Two hundred ninety‐two of these dogs were from Italy, additional 56 animals had been imported by Hungary, while 4 dogs had been illegally imported from an unknown country. None of these dogs had undergone euthanasia, since their death was caused by illness or accident, but clinical signs occurring intravitam were not reported. At post‐mortem examination, the analysed dogs showed catarrhal or hemorrhagic enteritis (*n* = 137), enlargement of the mesenteric lymph nodes (*n* = 79), pneumonia or other pulmonary lesions (*n* = 70), and meningeal and/or encephalic hyperaemia (*n* = 49). For 55 animals, post‐mortem findings were not reported or were not fitting with those observed in pCCoV‐infected dogs.

### 2.2 Nucleic acid extraction

Samples collected for molecular investigations were homogenized with phosphate‐buffered saline (PBS), and subsequently, RNA/DNA extraction was performed using the automated extractor QIAsymphony (Qiagen) and the QIAsymphony DSP Virus/Pathogen Kit (Qiagen), following the manufacturer's instructions.

### 2.3 Detection of viral pathogens

Tissues samples of dogs were submitted to molecular detection of the main viral pathogens of dogs, including CCoV (Decaro et al., 2013), CPV (Decaro, Elia, et al., 2005), CAdV‐1 and CAdV‐2, (Dowgier et al., 2016), canine distemper virus (CDV) (Elia et al., 2006), canid alphaherpesvirus type 1 (CaHV‐1) (Decaro et al., 2008) and rotaviruses (Zeng et al., 2008).

### 2.4 CCoV genotyping and subtyping

The detected CCoV strains were characterized by means of two distinct real‐time RT‐PCR assays, specific for the genotypes CCoV‐I and CCoV‐II, targeting a fragment of the S gene (Decaro et al., 2013).

Samples that tested positive for CCoV‐II were subjected to subtype‐specific CCoV‐IIa and CCoV‐IIb gel‐based RT‐PCR assays targeting the S gene (Table 1) (Decaro et al., 2013). The PCR products were detected using the TapeStation 2,200 (Agilent Technologies) according to the manufacturer's protocol.

**TABLE 1:** Oligonucleotides used for detection and characterization of CCoV strains

Test	Primer/probe	Reference	Sequence (5′−3′)	Sense	Position	Amplicon size	Specificity
Real‐time RT‐PCR	CCoV‐F	Decaro et al., 2004	TTGATCGTTTTTATAACGGTTCTACAA	+	6585−6611^a^	99 bp	CCoV‐I/II
	CCoV‐R		AATGGGCCATAATAGCCACATAAT	−	6660−6683^a^		
	CCoV‐Pb		FAM‐ACCTCAATTTAGCTGGTTCGTGTATGGCATT‐TAMRA	+	6620−6650^a^		
Real‐time RT‐PCR	CCoVI‐F	Decaro, Martella, et al., 2005	CGTTAGTGCACTTGGAAGAAGCT	+	478−499^b^	111 bp	CCoV‐I
	CCoVI‐R		ACCAGCCATTTTAAATCCTTCA	−	567−588^b^		
	CCoVI‐Pb		FAM ‐CCTCTTGAAGGTACACCAA‐TAMRA	+	508−526^b^		
Real‐time RT‐PCR	CCoVII‐F	Decaro, Martella, et al., 2005	TAGTGCATTAGGAAGAAGCT	+	6878−6897^a^	105 bp	CCoV‐II
	CCoVII‐R		AGCAATTTTGAACCCTTC	−	6966−6982^a^		
	CCoVII‐Pb		FAM ‐CCTCTTGAAGGTGTGCC‐TAMRA	+	6906−6922^a^		
RT‐PCR	20,179	Decaro et al., 2010	GGCTCTATCACATAACTCAGTCCTAG	+	320−345^a^345^b^ 12531−12556^c^	758 bp (CCoV‐IIa)	CCoV‐I/II
	INS‐R		GCTGTAACATAGTCATCATTCCAC	−	1054−1077^b^	499 bp (CCoV‐IIb)	CCoV‐IIa
	174–268		CAACATGTAACCTTTGTCTGTGATCTGC	−	13002−13029^c^		CCoV‐IIb

Abbreviations: FAM, 6‐carboxyfluorescein; TAMRA, 6‐carboxytetramethylrhodamine.

^a^ Oligonucleotide position is referred to the sequence of CCoV‐I strain 259/01 (GenBank accession number AF502583).

^b^ Oligonucleotide position is referred to the sequence of CCoV‐IIa strain Insavc‐1 (GenBank accession number D13096).

^c^ Oligonucleotide position is referred to the sequence of CCoV‐IIb strain 174/06 (GenBank accession number EU856362).

### 2.5 Molecular characterization of pCCoV and CPV

Lung samples from the pCCoV‐infected dogs were used for the molecular characterization of pCCoV. The spike protein gene (ORF2) of the putative pCCoV strains was sequenced and analysed using the protocol reported by Alfano et al. (2019). The sequences were analysed using BioEdit software package and the NCBI and EMBL analysis tools.

Samples that tested positive for CPV were further characterized by type‐specific minor groove binder probe assays (Decaro, Elia, et al., 2006; Decaro, Elia, et al., 2005; Decaro et al., 2007; Decaro, Martella, et al., 2006) and sequence analysis of partial VP2 gene (Buonavoglia et al., 2001).

### 2.6 Sequence analysis and phylogeny

CCoV sequences were manually edited and analysed using the Geneious platform (version 10.1.3) (Biomatters Ltd.). Nucleotide similarity with sequences deposited in the GenBank database was assessed using the BLAST (http://blast.ncbi.nlm.nih.gov/Blast.cgi) and FASTA (http://www.ebi.ac.uk/fasta33) tools with default values used to find homologous hits.

For the construction of phylogenetic trees, a multiple alignment of all target sequences was performed, using MAFTT Multiple Sequence Alignment software version 7 (Katoh & Standley, 2013) and Geneious software, using the neighbour‐joining method, with the p‐distance model, 1,000 bootstrap replicates, and, otherwise, the default parameters in Geneious (version 10.1.3).

### 2.7 Nucleotide sequence accession number

The nucleotide sequences of the analysed pCCoV strains were deposited in GenBank under the following accession numbers: MN086803 (98960‐1/2016); MN086804 (98960‐3/2016); MN086805 (98960‐4/2016); MN086806 (32712/1/2016); MN086807 (78870/2016); MN086808 (103480/2014); MN086809 (31975/2015); MN086810 (43891‐1/2016); MN086811 (43891‐2/2016); MN086812 (91507/2017); MN086813 (91510/2017); MN086814 (34879/2017); MN086815 (43022/2017); MN086816 (91529/2017); and MN086817 (34865/2017).

### 2.8 Data analysis

The comparison between positive and negative pCCoV dogs was carried out by examining the data with a chi‐squared test, considering the statistically significant values *p* < .05, using the IBM SPSS Statistics 25 software. The confidence interval (CI 95%) was calculated using the prevalence parameter estimate with the Excel software.

## 3 RESULTS

### 3.1 Detection of CCoV and pCCoV in autochthonous and imported dogs

CCoV RNA was detected in the gut of 76 (21.59%) out of 352 tested dogs, while 35 animals (9.94%), about half of the positive dogs, tested positive for CCoV‐IIa in internal organs by means of typing and subtyping molecular assays (Decaro et al., 2013), thus showing the presence of putative pCCoV strains in the infected dogs (Table 2). Of these 35 dogs, 6 lived in Italy and 29 had been imported from other countries (25 from Hungary and 4 illegally imported from an unknown country). The proportion of pCCoV‐infected dogs varies markedly between regions: we found 6 pCCoV‐infected dogs from Italy (6/292, 2%), 25 infected animals from Hungary (25/56, 45%) and 4 animals of unknown origin (4/4, 100%). At necropsy, apart from acute, sometime hemorrhagic gastroenteritis, most of the pCCoV positive dogs had displayed lesions suggestive of systemic involvement, including pneumonia and encephalitis (Table 2).

**TABLE 2:** Signalment of dogs tested positive to pantropic CCoV and detection of viral co‐infections

Pantropic CCoV‐positive dogs	Year	Prot. no.	Breed	Age	Country of origin	Life conditions	Gross lesions	Extraintestinal tissues positive to pCCoV	Co‐infecting viruses
1	2014	103480	Pomeranian	Y	Italy	Client‐owned	UKN	Heart, liver, spleen, lungs	CPV (2a), CDV
2	2015	31975	Mixed breed	Y	Italy	Stray dog	UKN	Heart, liver, spleen, lungs	CPV (2b)
3	2015	15910‐3	Cavalier king charles spaniel	Y	Hungary	Imported	Enteritis, splenomegaly, pneumonia, meningeal and encephalic hyperaemia	Brain, heart, liver, spleen, lungs, kidney	CPV (2a), CDV
4	2016	32712‐1	Mixed breed	Y	Italy	Stray dog	UKN	Brain, heart, liver, spleen, lungs, kidney	CPV (2b), CAdV‐1
5	2016	43891/1	Mixed breed	J	Italy	Stray dog	Enteritis, meningeal and encephalic hyperaemia	Brain, heart, liver, spleen, lungs	None
6	2016	43891‐2	Mixed breed	J	Italy	Stray dog	Enteritis, meningeal and encephalic hyperaemia	Brain, heart, liver, spleen, lungs	None
7	2016	78870	Chihuahua	Y	Italy	Client‐owned	UKN	Brain, heart, liver, spleen, lungs, kidney	CPV (2b), CAdV‐2, CDV
8	2016	98960‐1	English Bulldog	Y	UKN	Illegally imported	Enteritis, enlargement of mesenteric lymph nodes, pulmonary atelectasis and emphysema, meningeal hyperaemia	Brain, heart, liver, spleen, lungs	CPV (2a), CAdV‐2
9	2016	98960‐2	Pug	Y	UKN	Illegally imported	Enteritis, enlargement of mesenteric lymph nodes, pneumonia, meningeal and encephalic hyperaemia	Brain, heart, liver, spleen, lungs	CPV (2a), CAdV‐2
10	2016	98960‐3	English bulldog	Y	UKN	Illegally imported	Enteritis, enlargement of mesenteric lymph nodes, pneumonia and pulmonary oedema	Brain, heart, liver, spleen, lungs	CPV (2a), CAdV‐2
11	2016	98960‐4	Dogue de bordeaux	Y	UKN	Illegally imported	Enteritis, enlargement of mesenteric lymph nodes, pneumonia and pulmonary oedema, meningeal and encephalic hyperaemia	Brain, heart, liver, spleen, lungs	CPV (2a), CAdV‐2
12	2017	34865	Husky	Y	Hungary	Imported	Haemorrhagic enteritis, enlargement of mesenteric lymph nodes, meningeal and encephalic hyperaemia	Brain, heart, liver, spleen, lungs	None
13	2017	34873	Spitz	Y	Hungary	Imported	Haemorrhagic enteritis, enlargement of mesenteric lymph nodes, meningeal and encephalic hyperaemia	Brain, heart, liver, spleen, lungs	None
14	2017	34875	Spitz	Y	Hungary	Imported	Enteritis, enlargement of mesenteric lymph nodes, pneumonia	Brain, heart, liver, spleen, lungs	CPV (2a)
15	2017	34876	Spitz	Y	Hungary	Imported	Haemorrhagic enteritis, enlargement of mesenteric lymph nodes, pulmonary oedema	Brain, heart, liver, spleen, lungs	None
16	2017	34879	Shiba inu	Y	Hungary	Imported	Enlargement of mesenteric lymph nodes, meningeal hyperaemia	Brain, heart, liver, spleen, lungs	None
17	2017	42211	Shitzu	Y	Hungary	Imported	Enteritis, enlargement of mesenteric lymph nodes	Brain, heart, liver, spleen, lungs	CPV (2a)
18	2017	43020	Maltese	Y	Hungary	Imported	Enteritis, enlargement of mesenteric lymph nodes, pneumonia	Brain, heart, liver, spleen, lungs	CPV (2a)
19	2017	43021	Mixed breed	Y	Hungary	Imported	Enteritis, enlargement of mesenteric lymph nodes	Brain, heart, liver, spleen, lungs	CPV (2a, 2b)
20	2017	43022	Mixed breed	Y	Hungary	Imported	Enteritis, enlargement of mesenteric lymph nodes, pneumonia	Brain, heart, liver, spleen, lungs	CPV (2b), CaHV‐1
21	2017	91490	Spitz	Y	Hungary	Imported	Enteritis, enlargement of mesenteric lymph nodes, pneumonia, meningeal and encephalic hyperaemia	Brain, heart, liver, spleen, lungs	CPV (2a)
22	2017	91494	Spitz	Y	Hungary	Imported	Enteritis, enlargement of mesenteric lymph nodes, pneumonia, meningeal and encephalic hyperaemia	Brain, heart, liver, spleen, lungs	CPV (2a), CAdV2
23	2017	91498	Maltese	Y	Hungary	Imported	Enlargement of mesenteric lymph nodes	Brain, heart, liver, spleen, lungs	None
24	2017	91501	French Bulldog	Y	Hungary	Imported	Enteritis, enlargement of mesenteric lymph nodes	Brain, heart, liver, spleen, lungs	CPV (2a), CAdV2
25	2017	91504	Spitz	Y	Hungary	Imported	Enteritis, enlargement of mesenteric lymph nodes	Brain, heart, liver, spleen, lungs	CPV (2a), CAdV2
26	2017	91507	Spitz	Y	Hungary	Imported	Enteritis, enlargement of mesenteric lymph nodes, pneumonia, meningeal and encephalic hyperaemia	Brain, heart, intestine, liver, spleen, lungs	CPV (2a), CAdV2
27	2017	91510	Maltese	Y	Hungary	Imported	Enteritis, enlargement of mesenteric lymph nodes, pneumonia	Brain, heart, intestine, liver, spleen, lungs	CPV (2a), CAdV2
28	2017	91513	Yorkshire	Y	Hungary	Imported	Enteritis, enlargement of mesenteric lymph nodes, pneumonia, meningeal hyperaemia	Brain, heart, intestine, liver, spleen, lungs	CPV (2a), CAdV2
29	2017	91517	Maltese	Y	Hungary	Imported	Enteritis, enlargement of mesenteric lymph nodes, pneumonia	Brain, heart, intestine, liver, spleen, lungs	CPV (2a), CAdV1, CAdV2
30	2017	91519	Maltese	Y	Hungary	Imported	Haemorrhagic enteritis, enlargement of mesenteric lymph nodes, pneumonia	Brain, heart, intestine, liver, spleen, lungs	CPV (2a), CAdV2
31	2017	91529	Poodle	Y	Hungary	Imported	Enteritis, enlargement of mesenteric lymph nodes, pulmonary oedema	Brain, heart, intestine, liver, spleen, lungs	CPV (2a), CAdV2
32	2017	91531	Yorkshire	Y	Hungary	Imported	Haemorrhagic enteritis, enlargement of mesenteric lymph nodes, pneumonia	Brain, heart, intestine, liver, spleen, lungs	CPV (2a), CAdV2
33	2017	91538	Maltese	Y	Hungary	Imported	Enteritis, enlargement of mesenteric lymph nodes	Brain, heart, intestine, liver, spleen, lungs	CPV (2a), CAdV2
34	2017	91542	Maltese	Y	Hungary	Imported	Enteritis, enlargement of mesenteric lymph nodes	Brain, heart, intestine, liver, spleen, lungs	CPV (2a, 2b), CAdV2
35	2017	94583	Maltese	Y	Hungary	Imported	Enteritis, pneumonia	Brain, heart, intestine, liver, spleen, lungs	CPV (2a), CAdV2

Abbreviations: J, juvenile (6‐ to 12‐month‐old); UKN, unknown; Y, young (0‐ to 6‐month‐old).

### 3.2 Molecular characterization of putative pCCoV strains

Fifteen pCCoV strains were sequenced: 6 were from dogs of Italy and 9 from animals imported from other countries (6 from Hungary and 3 from an unknown country). The sequenced pCCoV strains presented neither the deletion in the genes of the accessory 3abc proteins nor the D125N mutation that had been suggested as potential markers for the pantropic behaviour (Decaro et al., 2008, 2013).

**FIGURE 1:**
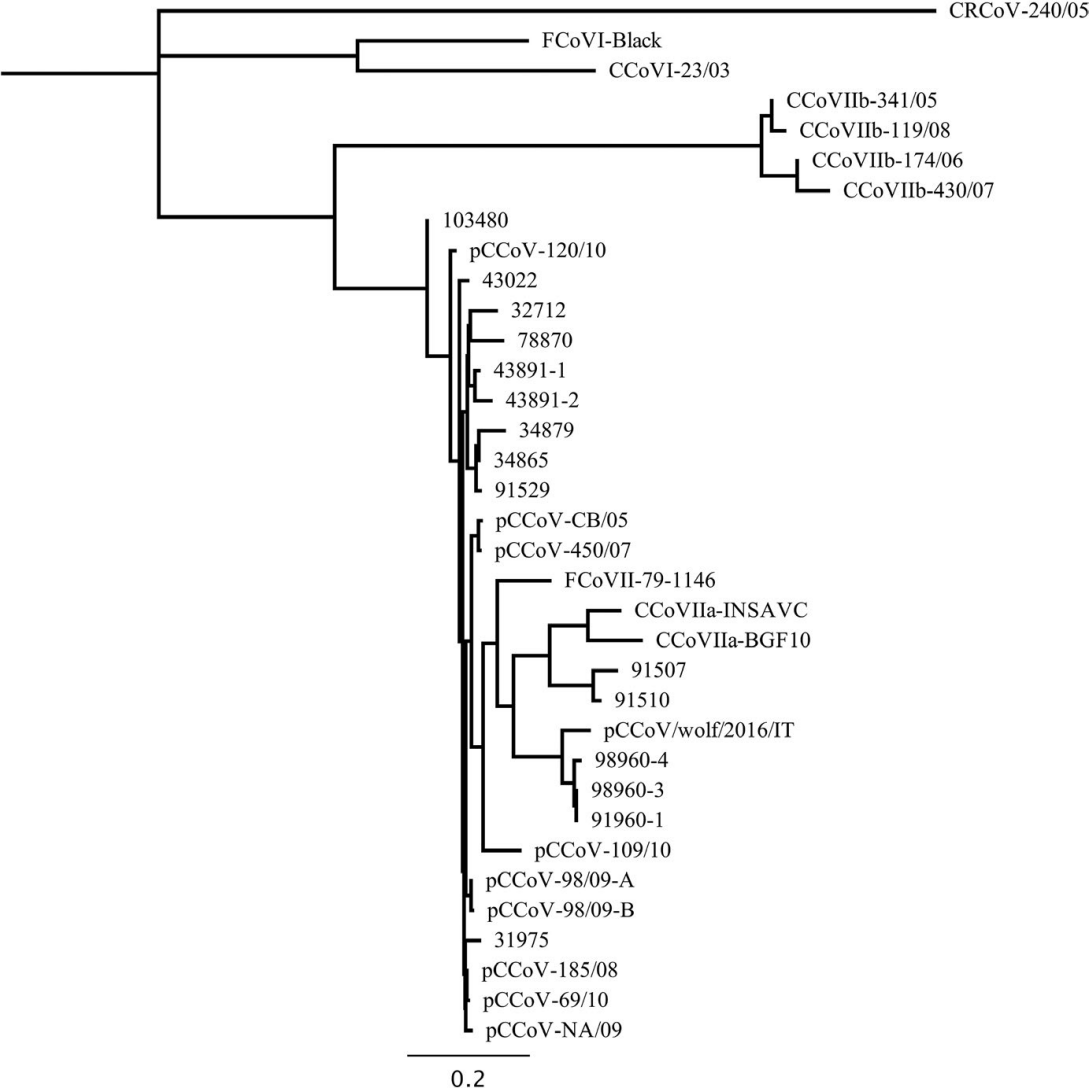
Phylogenetic tree generated with the neighbour‐joining method from partial spike protein gene (ORF2) sequences of the putative pantropic canine coronavirus strains and reference carnivore alphacoronaviruses

The phylogenetic tree, generated from partial ORF2 gene sequences, based on neighbour‐joining method (Figure 1) showed 5 different clustering groups. Strain 103480 felt in a cluster quite distant from all the others; 8 strains (32712, 78870, 43891‐1, 43891‐2, 34879, 43022, 91529, 34865) clustered with the Italian pCCoV strain 120/10; 2 strains (91507, 91510) were intermingled with enteric CCoVs; 3 other strains (98960‐1, 98960‐3, 98960‐4) clustered with strain pCCoV/wolf/2016/IT and 3 Asian enteric CCoVs. Another strain (31975) clustered with pCCoV strains identified in Italy and Greece (Ntafis et al., 2012).

### 3.3 Detection of viral co‐infections

Only 7 dogs (two adults and five pups) showed a single pCCoV infection, while the other 28 pCCoV‐infected animals displayed co‐infections caused by CPV, CDV, CAdV‐1, CAdV‐2, and CHV‐1 (Table 2). Pantropic CCoV/CPV infections were found in 6 animals, with 4 and 1 dogs being infected by CPV‐2a and CPV‐2b, respectively, whereas one animal was co‐infected by both CPV variants. Twenty dogs had triple infections caused by pCCoV, CPV and CAdV‐2 (*n* = 16); pCCoV, CPV and CAdV‐1 (*n* = 1); pCCoV, CPV and CDV (*n* = 2); and pCCoV, CPV and CHV‐1 (*n* = 1), and the remaining 2 dogs displayed quadruple infections caused by CCoV, CPV‐2, CAdV‐1 and CAdV‐2 (*n* = 1), or pCCoV, CPV‐2, CDV and CAdV‐2 (*n* = 1). The statistical association of co‐pathogens to pCCoV is shown in Table 3, which reports the detection rates of selected viral agents in pCCoV positive and negative samples. Both CPV and CAdV‐2 were significantly associated to pCCoV infection (*p* < .00001), whereas CAdV‐1 and CDV were not (*p* = .50 and *p* = .62, respectively).

**TABLE 3:** Prevalence of viral co‐pathogens in dogs with and without pCCoV infection

Virus	pCCoV positive (*n* = 35)		pCCoV negative (*n* = 317)		*p*‐value
	Positive (%)	95% CI	Positive (%)	95% CI	
CPV	28 (80.0%)	(0.693984–0.906016)	94 (30.0%)	(0.281621–0.311439)	**<.00001**
CAdV‐1	2 (5.7%)	(0.052749–0.061537)	11 (3.5%)	(0.034001–0.035399)	.504
CAdV‐2	18 (51.4%)	(0.429129–0.599442)	24 (7.6%)	(0.073505–0.077915)	**<.00001**
CDV	3 (8.6%)	(0.077765–0.093664)	36 (11.0%)	(0.109598–0.117531)	.618

^a^ Bold numbers indicate statistically significant *p* values (*p* < .05).

## 4 DISCUSSION

To date, pCCoV has been detected only sporadically in Italy and other countries (Buonavoglia et al., 2006; Chen et al., 2019; Decaro et al., 2012, 2013; Ntafis et al., 2012; Pinto et al., 2014; Zicola et al., 2012). In this study, we examined 352 dogs and identified 35 putative pCCoV‐infected animals, thus showing a wide distribution of this emerging virus. Twenty‐eight of the putative pCCoV‐infected dogs displayed double, triple or quadruple co‐infections with other viral pathogens. Therefore, in most cases, the identification of other pathogens in the same animals does not allow a clear association between the detected pCCoV strains and the death of the animals. Even for 7 dogs (two juvenile dogs and five pups), in which no associations with other viral pathogens were demonstrated, there is no definitive evidence that pCCoV was the cause of their death. In fact, previous studies have demonstrated that this virus is frequently associated to subclinical infections and impairment of the lymphocyte counts, rather than to severe clinical signs and death of the infected dogs (Marinaro et al., 2010). Most of the pCCoV‐positive animals also displayed post‐mortem findings accounting for a systemic involvement, but at which extent those lesions were induced by pCCoV or by other co‐pathogens, including the highly pathogenic CPV, CAdV‐1 and CDV, infecting the same dogs could not be assessed.

Remarkably, most of the pCCoV‐infected dogs had been recently imported from Hungary, which may account for a wider circulation of this virus in eastern Europe. This finding is in contrast with those of a previous study aiming to assess the pCCoV circulation in Europe, which reported similar prevalences in Italy and Hungary, with detection rates of 8.69% and 9.33%, respectively (Decaro et al., 2013).

Currently, no test is available to differentiate the pantropic from the enteric CCoV strains, since no specific genetic markers have been identified in pCCoV so far. As a consequence, only the detection of a CCoV‐IIa strain in extraintestinal tissues accounts for a possible pCCoV infection in dogs. This situation is similar to that observed in calicivirus infections in cats, where markers of pathogenicity have been not yet detected in highly virulent strains, so that diagnosis of systemic calicivirosis is obtained when the virus is detected in extrarespiratory tissues (Caringella et al., 2019).

Different from coronavirus infections in dogs, in cats potential genetic signatures were recently detected, which are able to discriminate between feline infectious peritonitis and feline enteric coronavirus strains (Felten et al., 2017).

According to previous observations (Decaro et al., 2013), two of the putative pCCoV strains identified in this study (91507 and 91510) fell in the same cluster with enteric CCoV strains, while other strains (103480, 32712, 78870, 43891‐1, 43891‐2, 34879, 43022, 91529, 34865, 91507, 91510, 31975) were more closely related to pCCoVs reference strains. Surprisingly, 3 pCCoVs (98960‐1, 98960‐3, 98960‐4) clustered with viruses detected in wildlife or domestic cats in Asia (Wang, Ma, Lu, & Wen, 2006). These strains were from dogs illegally imported from an unknown country, which highlights the role of illegal trade of dogs in the introduction of pathogens into Italy (Decaro et al., 2007; Mira et al., 2018).

An additional finding of the present study is the high frequency of co‐infections with pCCoV and other viruses. Enteric CCoV infections in dogs are very frequent (Decaro & Buonavoglia, 2008; Decaro et al., 2008; Pratelli et al., 2003; Priestnall, Pratelli, Brownlie, & Erles, 2007). Co‐infection by CPV and CCoV in dogs is known to enhance the severity of clinical signs (Decaro, Elia, et al., 2006; Evermann, Abbott, & Han, 2005; Pratelli, 2006), with fatal outcomes being frequently reported in pups (Decaro, Elia, et al., 2006). A high frequency of co‐infections with pCCoV and other pathogens has been previously reported (Alfano et al., 2019; Decaro et al., 2013; Ntafis et al., 2012; Pinto et al., 2014; Zicola et al., 2012). In the present study, we found a significant association of pCCoV with CPV and CAdV‐2 infections. Pantropic CCoV is able to affect lymphocyte counts, thus causing a prolonged lymphopenia, so that it has been postulated that pCCoV infection may predispose to the increase in virulence of other pathogens by inducing immunosuppression in the infected dogs (Marinaro et al., 2010).

The large population of unvaccinated free‐ranging dogs present in Italy (Corrain et al., 2007; Verardi, Lucchini, & Randi, 2006) considerably increases the density of susceptible hosts, and may thus importantly impact the spread and maintenance of canine pathogens in the environment. Therefore, it is strongly recommended to vaccinate not only private‐owned dogs but also stray dogs whenever possible. In addition, the epidemiological risk related to the legal and illegal trade of carnivores from Asian countries must be taken into account, since this trade may represent a source of several emerging pathogens in domestic and wild canids (Mira et al., 2018, 2019).

## 5 CONCLUSION

The present study demonstrates an increasing circulation of pCCoV in Italy, which reinforces the need for intensive and continuous surveillance on the importation and illegal trade in animals and the need for increased controls on both autochthonous and imported dogs.

## ETHICAL APPROVAL

Ethical statement is not applicable since sample collection was obtained from dead animals that were submitted to diagnostic investigations upon request of the owners or public authorities.

## CONFLICT OF INTEREST

The authors declare that they have no conflict of interest.

## Data Availability

The data that support the findings of this study are openly available in the GenBank database at https://www.ncbi.nlm.nih.gov/nucleotide/ under accession numbers MN086803‐MN086817.
